# Afghan Agenda in Current Canadian Politics

**DOI:** 10.1134/S1019331622080044

**Published:** 2022-06-29

**Authors:** E. V. Issraelyan

**Affiliations:** Institute for the U.S. and Canadian Studies, Russian Academy of Sciences, Moscow, Russia

**Keywords:** **Кеywords:** Canadian foreign policy, federal elections, Afghanistan, Taliban, refugee admission, Liberal Party of Canada, Justin Trudeau, Conservative Party of Canada, New Democratic Party of Canada, United Nations High Commissioner for Refugees (UNHCR)

## Abstract

Canada’s operation in Afghanistan has been unfolding during a critically important domestic political event, i.e., the federal elections of 2021. The election campaign had pooled the main attention and resources of the Canadian leadership, limiting its ability to act in Afghanistan. Despite the difficulties, the Liberal Government of Justin Trudeau has achieved a lot. Firstly, they have organized the evacuation of Canadians and of Afghans who worked with the Canadian Armed Forces during the US and NATO military mission. Secondly, Ottawa has defined its attitude towards the Taliban regime by refusing diplomatic recognition. Thirdly, the admission of Afghan refugees to Canada has begun. In each of these areas, the Liberal Government has successes and failures, which have caused acute controversy in the country.

## INTRODUCTION

The 2021 election campaign in Canada coincided with two mutually related events having a strong international resonance: firstly, the seizure of power by the Taliban in Afghanistan and, secondly, the completion of the withdrawal of American troops from this country. Amid reports about the election battles, the Canadian media regularly published chilling accounts of desperate attempts by Afghan nationals to flee their country, conquered by the Taliban.

Ottawa was directly involved in the Afghan events. Canadians took the terrorist attacks of September 11, 2001 in the United States as a blow to all Western democracies and to their country in particular, especially since over 30 Canadians died that day at the World Trade Center in New York. Political figures in Canada repeatedly raised the idea of retaliation for the dead by participating in the 2001–2011 Afghan mission.^1^ Canada, like other Western countries, saw Afghanistan as a platform for practicing methods of combating terrorism. Stephen Harper’s government (2006–2015) made a decision on Canada’s participation in the US and allied operation in Afghanistan.

This operation has become Canada’s largest military campaign since the Korean War of 1950–1953. In terms of duration (almost ten years), this operation has exceeded the country’s participation in the First and Second World Wars combined (Issraelyan and Evtikhevich, 2013, pp. 145–170).

Canada was among the first countries to contribute its armed forces and civilian advisers in the fall of 2001 to support the US counterterrorism operation. In 2003–2004, Canada, along with the United States, played a decisive military role on this axis, and the Canadian contingent accounted for about 40% of all International Security Assistance Forces (ISAF) (Volodin, 2007, p. 44). The operation required large-scale financial resources. About CA$ 18.5 billion were spent on it (Afghanistan in Review, 2021). Grievous statistics should be given too: 165 Canadians died in that campaign and about 2000 were injured. Moreover, most of the Canadian military personnel were concentrated in Kandahar, the most dangerous province with the largest concentration of the Taliban.

In addition to military participation in the Afghan operation, Canada was one of the top five donors supporting the so-called *nation building* in Afghanistan, taking the lead in education investment. It should be noted that in those years, Afghanistan was the main recipient of Canadian development assistance, $3.6 billion from 2001 to 2011 (Mank, 2021). The focus of Canadian assistance projects was on protecting women’s rights and promoting gender equality.

The withdrawal of US troops from Afghanistan, which ended in August 2021, opened a new page in Canada–Afghanistan relations. The situation in Afghanistan required quick decisions from Ottawa, provoked controversy among the participants in election debates, and made the media headlines.

## END OF THE US MILITARY MISSION: NEW ISSUES FOR CANADA

By August 31, 2021, the armed forces of the United States and its allies left Afghanistan, drawing a line under their 20-year military presence in this country. The withdrawal of American troops began under President Barack Obama (2009–2017) and continued under Donald Trump (2017–2021). This operation was finally wound up under the administration of Joe Biden (in power since January 20, 2021), who repeatedly announced the terms and conditions for ending the Afghan operation. Despite the lengthy process and the numerous tips-off from American officials, Canada found itself unprepared for the new challenges in the Afghan agenda of its foreign policy.

The Taliban seized power on August 15, the day Canada announced federal elections to the parliament. Prime Minister Justin Trudeau faced the difficult task of working on two fronts: the Afghan and domestic political agenda. Each area required a large concentration of human, material, and organizational resources. Information was leaked to the press that Trudeau attempted to postpone the withdrawal of Canadian troops from Afghanistan but received a categorical refusal from Washington [10]. Canada had to act exactly according to the American timetable. At the request of the United States, Canada stopped the evacuation of civilians from Afghanistan the day before the official deadline to free up airspace for American aircraft.

Observers also pointed out another fact. On the eve of the Group of Seven (G7) Summit in August 2021, Biden personally consulted on the Afghan issue with the leaders of the closest NATO allies: Britain, Germany, France, and Italy. Canada was not part of the discussions, another sign of its diminishing role in international affairs and in the US–Canada dialogue.

The main issue Ottawa faced when the Taliban came to power in Afghanistan was the evacuation of Canadians and of those Afghan nationals who worked with Canadian military and civilian agencies. In a statement signed by three Canadian ministers—Foreign Affairs; National Defense; and Immigration, Refugees, and Citizenship—the government condemned the escalating violence in Afghanistan, especially against women, girls, and ethnic minorities. It also announced a suspension of the embassy’s operations and promised to ensure the admission and safety of the Afghans who cooperated with Canadian representatives [3]. The embassy staff immediately left Afghanistan.

Trudeau signed the resolution adopted at the end of August 2021 by the heads of more than 90 countries worldwide on coordinated efforts to create conditions for the unhindered exit from Afghanistan of their countries’ nationals and of Afghan nationals of two categories: those who were part of the risk groups and those who assisted Western countries in fulfilling their mission. This classification set the framework for the Canadian admission policy for immigrants and refugees from Afghanistan.

Unlike some other top public officials who considered the possibility of interaction with the Taliban, Trudeau immediately announced that his administration did not plan to recognize the new government of Afghanistan. He recalled that Canada refused to interact with the former Taliban regime, which was in power in 1996–2001, having declared their movement a terrorist organization [2].

The Prime Minister’s stance caused a mixed response in Canada. Most of the political elite and the public supported the diplomatic boycott of the Taliban and objected to their removal from the list of terrorist organizations. However, advocates of a different approach also came forth. Thus, Maryam Monsef, a Canadian politician of Afghan origin, who held various positions in Trudeau’s cabinet, called the Taliban “our brothers” during an official briefing. Monsef has paid for her statement—many observers believe it was the real reason why she lost in her own constituency at the 2021 elections.

Dissenting points of view also exist in the academic community. Some insist on working with the Taliban, including Colin Robertson, a former diplomat, now Vice President of the country’s leading think tank—the Canadian Global Affairs Institute. He believes that diplomatic recognition should not be seen as a “seal of approval” of the Taliban policies, but rather as a means to protect and advance Canada’s national interests. Firstly, Canada has invested heavily in Afghanistan in terms of material and human resources, and now, in tandem with the United States and other allies, Canada must help the new government maintain the achievements of the previous years and continue the unfolding reforms. Secondly, the evacuation from Afghanistan is not complete yet, and the success of this process hinges on the interactions with the Taliban. Thirdly, Ottawa needs to work with the Taliban to achieve its main foreign policy goal, i.e., restoring the role and influence of Canada on the world arena. One of its priorities is to expand Canada’s “presence on the ground” in different parts of the planet. Canada has experience in developing diplomatic relations with “unfriendly regimes,” e.g., the recognition of China in 1970 and the renunciation of the trade, economic, and diplomatic boycott of Cuba in 1961. After establishing relations with the Taliban, Canada should resume diplomatic relations with North Korea and Iran, sums up Robertson [12].

The Canadian Air Force has finished its operation to evacuate people from Afghanistan, having performed 17 flights and bringing about 3700 Canadians and Afghans to the Canadian territory. The newcomers were provided accommodations in British Columbia, Alberta, Manitoba, Ontario, New Brunswick, and Prince Edward Island. In addition, Ottawa committed to take in 5000 Afghan refugees in the near future, who were evacuated by American aircraft to the United States or to US and NATO military bases in other countries. For reference: these bases are located in Qatar, Bahrain, Kuwait, the United Arab Emirates (UAE), Saudi Arabia, Germany, Spain, and Italy; at these locations, the Afghans go through security checks before being taken to the United States.

There are two important promises made by Ottawa. In line with the “sunny ways” policy, based on humanitarian and liberal values, which was the hallmark of Trudeau’s course in previous years, the government declared commitment to accept 20 000 Afghan refugees. The Liberal Party’s platform mentioned 20 000 to 40 000 Afghan migrants. Marc Garneau, the Minister of Foreign Affairs of Canada, announced at the UN the finally agreed number of 40 000 people [1]. In addition, Ottawa provided $50 million in humanitarian aid [8].

This humanitarian message won the approval of nongovernmental organizations involved in protecting the rights of immigrants and refugees. Meanwhile, the rushed campaign to evacuate civilians from Afghanistan, amid chaos and confusion, left many questions unanswered. One such question is how the repatriation of Canadian citizens remaining in Afghanistan will take place. According to official data, as of September 2021, more than 1200 Canadians, their families, and persons with a residence permit in Canada stayed on the territory of Afghanistan [8]. When questioned, officials got away with vague statements about coordinated efforts with their allies to ensure the security and safety of civilians and save the lives of Afghans.

Assessing the results of the operation to evacuate civilians who were willing to leave Afghanistan, it should be noted that Canada was not among the leaders in terms of the number of evacuated civilians (i.e., the key indicator). According to Reuters [15], as of August 30, 2021, the United States was ranked first according this indicator by a wide margin (114 000). Qatar and the UAE helped evacuate, respectively, more than 40 000 and about 36 500 people. Among the G7 countries, Canada (3700) was ahead of only France (3000) and Japan (less than 500 people), falling behind Britain (315 000), Germany (5347), and Italy (5011).

## AFGHAN ISSUE IN THE 2021 ELECTION CAMPAIGN FOR THE PARLIAMENT OF CANADA

Let us revisit to the issues around the early parliamentary elections in Canada. It is known that Canadians, as one journalist put it figuratively, “vote with their wallets.” That is to say, they focus on the socio–economic and financial aspects of party programs, without taking much interest in their foreign policy aspects. The events in Afghanistan somewhat changed this unwritten rule. Although the COVID-19 pandemic remained the central theme of the election battles, foreign policy issues also played a prominent role. Together with Afghanistan, debates focused on the development prospects of Canada–China relations, the fight against global warming, environmental protection, and the protection of sovereignty in the Arctic.

The Afghan operation stood as a separate issue during the debates of political party leaders in September 2021: Justin Trudeau (Liberal Party), Erin O’Toole (Conservative Party), Jagmeet Singh (New Democratic Party), Yves-François Blanchet (Bloc Québécois), and Annamie Paul (Green Party). Unlike the 2015 elections, when the divide between the parties ran on the number of Syrian refugees being admitted, the 2021 debates centered on the lessons and shortcomings of the evacuation campaign itself. Understandably, Trudeau focused on the achievements of Canadian military and civilian personnel, who managed to save the lives of thousands of people.

All other politicians unanimously criticized the government for the untimely elections. O’Toole accused Trudeau of “political selfishness” and unwillingness to abandon the power struggle for the sake of ending violence and ensuring the safety of the Afghans. Furthermore, O’Toole and Singh reproached the government for sluggishness, poor organization of the campaign, lack of coordination in the actions of the various ministries and departments, and bureaucratization of the refugee status application processing. Paul, the Green Party leader, pointed out shortcomings in the operations of the intelligence service, noting sarcastically: “It seems like we got better information on our smartphones than Mr. Trudeau got from our entire intelligence service” [4].

Opinion polls indicated that Canadians were generally dissatisfied with the government’s efforts to evacuate civilians from Afghanistan. According to the Angus Reid Institute, the number of respondents who called the government’s actions “successful” was close to zero (2%); 37% considered the operation “a failure”; 20% restrained from judgment; and 41% of those surveyed said that the operation went “as well as can be expected.” This view was shared by the electorate of the Liberal Party, the New Democratic Party, and the Bloc Québécois, while 37% of Canadians supporting the Conservative Party called the operation “a failure.” An indicator of the generally negative attitude among the public towards government policy is the opinion expressed by half of the respondents that Canada should leave Afghanistan permanently [9].

Dissatisfaction with the process and outcomes of the evacuation from Kabul was elicited in the results of another survey. It was conducted by Nanos Research and found that 45% of respondents rated the government performance “poor” or “very poor” (17% and 28%, respectively). Men tend to be more disapproving of the country’s leadership than women; i.e., 52% of the men and 38% of women surveyed believe that the government did a “poor” or “very poor” job [11].

Criticism and harsh judgments of Trudeau were undoubtedly justified. The evacuation campaign was indeed fraught with organizational blunders, strategic mistakes, and attempts to shift obligations to partners (as was the case with the Ukrainian Air Force, which helped evacuate about two dozen Canadian and Afghan nationals first to Kiev and then to Canada).

However, it is also true that Trudeau has achieved much. First of all, in the number of people evacuated, i.e., a quantitative indicator of the operation performance, Canada was ahead of many NATO members, including, as mentioned above, two G7 members. Moreover, Canada operated in very difficult conditions. Firstly, it was one of the first countries to cease its participation in the NATO military operation in Afghanistan. After that, the ties between Canada and Afghanistan weakened, and the Afghan issue virtually disappeared from Ottawa’s political agenda. As a result, the Canadian military worked in an unfamiliar environment during the evacuation. Secondly, it so happened that the Afghan operation unfolded during a major domestic political event in Canada, i.e., federal elections. They pooled the main attention and resources of the Canadian leadership. All these factors should be taken into account when analyzing the achievements and costs of Trudeau’s mission in Afghanistan.

## AFGHAN REFUGEE ADMISSION POLICY

The Ministry of Immigrants, Refugees, and Citizenship developed two programs to receive Afghan migrants. The first one was called the Immigration Program for Afghans Who Assisted the Government of Canada [6]. To be eligible, an applicant must meet the following criteria:

Firstly, the applicant must be an Afghan national who worked with the Government of Canada (as an interpreter who provided services to the Canadian Forces or as a local staff at the Embassy of Canada) and had to be in Afghanistan on or after July 22, 2021, i.e., the date the immigration program started. However, it was indicated that even if this requirement was not observed, the applicant could still expect his/her documents to be processed. The applicant must also be admissible to Canada, and the program guidelines explained in detail when someone could be found *persona non grata* by the immigration authorities of Canada. Three reasons were listed: security reasons (suspicion of espionage, subversion, violence or terrorism, participation in other criminal activities); medical reasons; financial reasons (inability/unwillingness of the applicant to support him/herself and family members). “Committing a crime, including driving while under the influence of drugs or alcohol” was indicated separately as a reason for inadmissibility. The program also covered the applicant’s family members: a spouse or common-law partner; a dependent child (grandchild) who must not be married or in a common-law relationship. The age requirement for the dependents was under 22 years. As of November 17, 2021, Canadian immigration services had registered 14 520 applications under this program; of these, 5000 were approved, and another 3460 Afghans entered Canada with refugee status [13].

The second program is humanitarian in nature as it provides an opportunity for certain categories of Afghan nationals to resettle in Canada [5]. It covers the following Afghan nationals outside of Afghanistan: women leaders, human rights activists, representatives of persecuted ethnic and religious minorities, LGBT communities, journalists, and people who assisted Canadian journalists. To be eligible for resettlement in Canada, the applicant must comply with many formalities. Potential settlers may enter Canada under a government program or a private sponsorship program, each of which is valid for a year. Contacting directly the Ministry of Immigrants, Refugees, and Citizenship is not allowed. The applicant must first register for refugee status with the United Nations High Commissioner for Refugees (UNHCR) or the Immigration Service of the host country. Then, his/her documents are sent to Canada by one of these agencies or by an authoritative international public organization with which the Canadian government cooperates. The document package submitted under the private sponsorship program must also include an agreement signed by the sponsor on his/her commitments, including an attachment with a thorough list of services and expenses provided by the sponsor. Not surprisingly, due to the bureaucratic obstacles, only 400 people have been able to resettle in Canada under the humanitarian program [13].

The humanitarian component of the Afghan operation was a continuation of Trudeau’s government’s immigration policy of previous years. It was recognized by the international community and experts as one of the most successful areas of the government work. The Liberals in power amended the Canadian Citizenship Act to expand the rights of migrants and refugees and brought the illegal migration from the United States under control. In 2018, Canada accepted a record number of Syrian refugees—instead of the promised 25 000 migrants from Syria by 2021, Canada provided asylum to 75 000 people, ranking first in this indicator worldwide [7].

Canadian private sponsorship programs are particularly well known. In 1979, Canada became the world’s first country to “partially privatize” the admission of refugees. Under Canadian law, individuals, families, or groups of people can sponsor refugees and personally integrate them. In doing so, the government takes into account the sponsors’ choice regarding refugees. The sponsors, in turn, get used to living and communicating with people that represent a different culture. This practice allows Canadians to feel involved in political and global processes, gives them the opportunity to control immigration, and helps them fulfill their life purpose. Private sponsorship has reached such a scale in Canada that it has actually pushed the relevant state programs into the background. In 2019, only a third of the migrants received state assistance. The rest settled through the support of individuals or public organizations. The Canadian model of using private sponsorship in refugee admission has been rated highly by the UNHCR Office and other international structures. This practice formed the core of similar or fully analogous projects developed by France, Germany, New Zealand, and Spain (Van Haren, 2021).

The government policy of accepting Afghan refugees, as well as the evacuation campaign from Afghanistan, has not escaped criticism, which culminated in a letter from authoritative political and public figures of Canada, who addressed Trudeau and the ministers of the leading foreign affairs agencies. The letter was signed by well-known politicians such as the former Foreign Minister Lloyd Norman Axworthy and Senator Ratna Omidvar, prominent scholars (including Prof. Fen Hampson), and representatives of major human rights organizations. While recognizing the merits of the Afghan admission programs, the authors of the letter pointed to serious shortcomings and recommended measures to eliminate them. In particular, they proposed to (1) “clarify Canada’s policy by defining its terms” (according to the authors, the following terms need to be defined: “assistance to Canada” and “accepted categories” of persons under the humanitarian program); (2) “devote significant resources needed to get the job done,” including extra human resources for processing the applications submitted by Afghans; and (3) “waive the requirement of UNHCR recognition, and recognize the Afghan crisis as a prima facie refugee situation” [14]. The UN documents provide for such a procedure—in emergency circumstances when a group of migrants is granted refugee status, each member of this group automatically receives this status. The use of the prima facie approach instead of granting the refugee status on an individual basis makes it possible to eliminate many bureaucratic requirements and significantly reduce the time for processing applications.

## CONCLUSIONS

The Afghan agenda of Ottawa’s foreign policy became a national priority of Canada after the Taliban seized power in Kabul in August 2021. The situation in Afghanistan required urgent action from the government, which organized the evacuation of Canadians and of Afghans who assisted the Canadian Armed Forces during the US and NATO military missions. Ottawa defined its attitude towards the Taliban regime by refusing diplomatic recognition. Canada began to accept Afghan refugees on its territory. In each of these areas, Trudeau’s government had successes and failures, which caused acute controversy in Canada.

The domestic political context associated with the events in Afghanistan was out of the ordinary for Canada since the Afghan campaign coincided with the early federal elections to the Parliament of Canada. This whole situation, along with Canada–China relations, was vigorously debated during the election campaign. Judging by the public opinion polls, the events in Afghanistan, i.e., the withdrawal of American and NATO forces and the lightning-fast victory of the Taliban over the Afghan regular army, had a significant impact on the course of the election campaign. Trudeau had to address all these issues when determining the necessary actions and developing a policy since the Afghan agenda highlighted Ottawa’s foreign policy shortcomings, i.e., the decline in Canada’s significance in the world arena and in the US–Canada dialogue. These are the issues to be addressed by the third government of Justin Trudeau, who won the 2021 elections.

## SOURCES

1. Address by the Honourable Marc Garneau at the General Debate of the 76th United Nations General Assembly – “In Our Hands,” Global Affairs Canada, Sep. 27 (2021). https://www.canada.ca/en/global-affairs/news/2021/09/address-by-the-honourable-marc-garneau-pc-mp-minister-of-foreign-affairs-of-canada-at-the-general-debate-of-the-76th-united-nations-general-assembl.html. Cited October 20, 2021.

2. Bensadoun, E., Canada has ‘no plans’ to recognize Taliban as Afghanistan government, Trudeau says, Global News. Aug. 17 (2021). https://globalnews.ca/news/8118905/canada-will-not-recognize-taliban/. Cited September 20, 2021. 

3. Canada temporarily suspends operations at Embassy of Canada to Afghanistan, Global Affairs Canada Statement, Aug. 15 (2021). https://www.canada.ca/en/global-affairs/news/2021/08/html. Cited August 21, 2021.

4. Draper, S., Federal Election 2021: The unexpected role of foreign policy, National, Sep. 16 (2021). https://www.national.ca/en/perspectives/detail/federal-election-2021-the-unexpected-role-of-foreign-policy/. Cited October 16, 2021.

5. Humanitarian program for Afghan nationals in need of resettlement. Government of Canada. https://www.canada.ca/en/afghanistan/special-measures/about-humanitarian-program.html. Cited October 20, 2021.

6. Immigration program for Afghans who assisted the Government of Canada. Government of Canada. https://www.canada.ca/en/immigration-refugees-citizenship/services/refugees/afghanistan/special-measures/immigration-program.html. Cited November 24, 2021.

7. Kalata N., Syrian refugees reflect on a decade of war and their new lives in the Toronto area, CBC News, Mar. 15 (2021). https://www.cbc.ca/news/1.5947946. Cited July 15, 2021.

8. More than 3000 Evacuees Arrive in Canada from Afghanistan. Immigration, Refugees and Citizenship, Sep. 3 (2021). https://www.canada.ca/en/immigration-refugees-citizenship/news/2021/09/more-than-3000-evacuees-arrive-in-canada-from-afghanistan.html. Cited October 17, 2021.

9. Most say Canada should accept at least 20 000 Afghan refugees. Angus Reid Institute, Aug. 30 (2021). https://angusreid.org/afghanistan-evacuation-federal-election. Cited October 1, 2021.

10. View: Canada’s national humiliation in Afghanistan, National Post, Aug. 28 (2021). https://nationalpost.com/news/politics/election-2021/np-view-canadas-national-humiliation-in-afghanistan. Cited August 30, 2021.

11. Neustaeter B., Nearly half of Canadians say Liberal government did a poor job on Afghanistan evacuations: Nanos, CTV News, Sep. 3 (2021). https://www.ctvnews.ca/politics/1.5573006. Cited September 23, 2021.

12. Robertson C., Practicing good diplomacy will require Canada to recognize the Taliban in Afghanistan, The Globe and Mail, Aug. 31 (2021). 

13. Supporting Afghan nationals: Key figures. Government of Canada. Nov. 17 (2021). https://www.canada.ca/en/immigration-refugees-citizenship/services/refugees/afghanistan/key-figures.html. Cited November 20, 2021.

14. Urgent Call to action in response to the crisis in Afghanistan. Letter. Nov. 1 (2021). https://wrmcouncil.org/news/letter-news/urgent-call-to-action-in-response-to-the-crisis-in-afghanistan/. Cited November 25, 2021.

15. Factbox: Evacuations from Afghanistan by country, Reuters, Aug. 30 (2021). https://www.reuters.com/world/evacuations-afghanistan-by-country-2021-08-26. Cited November 25, 2021.

## Footnotes

1 Canada participated in the US counterterrorism operation in Afghanistan from 2001 and then as part of the International Security Assistance Force (ISAF) until the end of July 2011, when the main part of Canadian troops were withdrawn from this country. Then, the focus of Canada’s military activities shifted to training Afghan military personnel and policemen. In March 2014, the Canadian Armed Forces were completely withdrawn from Afghanistan.
